# Variation in the use of infection control measures and infection-related revision incidence after breast implant surgery in the Netherlands

**DOI:** 10.1016/j.jpra.2022.10.004

**Published:** 2022-10-12

**Authors:** Babette E. Becherer, Perla J. Marang-van de Mheen, Danny A. Young-Afat, Rene R.J.W. van der Hulst, Xavier H.A. Keuter, Hinne A. Rakhorst, Marc A.M. Mureau, Dutch Breast Implant Registry (DBIR) group.

**Affiliations:** a Department of Plastic and Reconstructive Surgery, Erasmus MC Cancer Institute, University Medical Center Rotterdam, Rotterdam, The Netherlands; b Dutch Institute for Clinical Auditing, Leiden, The Netherlands; c Department of Medical Decision Making, Leiden University Medical Center, Leiden, The Netherlands; d Department of Plastic and Reconstructive Surgery, Amsterdam University Medical Centers, location VUmc, Amsterdam, The Netherlands; e Department of Plastic and Reconstructive Surgery, Maastricht University Medical Center, Maastricht, The Netherlands; f Department of Plastic, Reconstructive and Hand Surgery, Medisch Spectrum Twente, Enschede, The Netherlands

**Keywords:** breast implant surgery, breast reconstruction, breast augmentation, infection control measures, infection-related revision surgery

## Abstract

**Background:**

The use and effect of most infection control measures (ICMs) in breast implant surgery are still debated, likely resulting in undesired variation in current practices.

**Objectives:**

This study investigated the relationship between the number and combinations of ICMs used and the infection-related revision incidence after breast implant surgery. Additionally, national variation between Dutch healthcare institutions in ICM use was evaluated.

**Methods:**

For this multicentre, population-based study, all patients who received a primary breast implant or tissue expander for breast augmentation or reconstruction between 2015 and 2019 were identified from the Dutch Breast Implant Registry. Seven prospectively collected ICMs were investigated: preoperative antibiotics, implant and/or pocket irrigation, glove change, nipple guards, insertion sleeve, postoperative drains, and postoperative antibiotics.

**Results:**

This study included 52,415 implants (85% augmentation, 15% reconstruction).The median (IQR) number of ICMs used was 3 (3-4) for augmentation and 4 (4-5) for reconstruction. Median follow-up was 30 months for augmentation and 34 months for reconstruction. Infection-related revision incidence was 0.1% for augmentation and 2.1% for reconstruction. Most infection-related revisions occurred within 2 months for augmentation and 2.5 months for reconstruction. The impact of ICM use on infection-related revision incidence remained unclear, given its low incidence. A significant variation was observed between institutions in the use of postoperative antibiotics and drains.

**Conclusions:**

Although the use of different ICMs varied considerably between institutions, the infection-related revision incidence after breast implant surgery was generally low. Most surgeons used four ICMs for breast reconstruction and three ICMs for breast augmentation. Further studies on the causes and effects of the observed variation are needed.

## Introduction

Breast augmentation is the most common cosmetic surgery performed worldwide. Breast reconstruction is one of the most commonly performed reconstructive procedures in plastic surgery.^1^, ^2^, ^3^ In the Netherlands, approximately 1 in 30 women has one or two breast implant(s).^4^ The majority of these women (85%) received breast implants for cosmetic augmentation, while 15% received breast implants for breast reconstruction.^5^^,^^6^

Unplanned revision of a breast implant or tissue expander is one of the most severe complications following breast implant surgery. Reported incidences are up to 3% after a cosmetic augmentation and up to 6% in patients after breast reconstruction.^7^, ^8^, ^9^, ^10^ Several studies have identified the potential predictors or risk factors for postoperative complications of breast implant surgery.^7^, ^8^, ^9^^,^^11^, ^12^, ^13^, ^14^, ^15^, ^16^ These predictors or risk factors, however, cannot always be controlled by surgeons or are not always associated with the quality of care delivered (e.g., radiotherapy or smoking). Therefore, to further reduce the unplanned revision incidence, clinicians need information on factors associated with these revisions that can be modified in daily clinical practice.

Many unplanned revisions are related to surgical site infections.^9^^,^^12^^,^^17^ Therefore, perioperative measures that could potentially result in fewer infections are of particular interest. Various guidelines advise administering prophylactic antibiotics before incision.^18^, ^19^, ^20^, ^21^ However, especially in the current era of evidence-based medicine, the beneficial effects of other measures such as postoperative antibiotics or nipple guards are still under debate.^20^^,^^22^, ^23^, ^24^, ^25^, ^26^ Therefore, the first aim of the present study was to investigate the association between the number and combinations of infection control measures (ICMs) used per breast implant insertion procedure and the infection-related cumulative revision incidence over time. The second aim was to investigate the national variation between Dutch healthcare institutions in the use of each ICM.

## Methods

### Design and study population

This observational cohort study included all women who had been prospectively registered in the Dutch Breast Implant Registry (DBIR) after receiving at least one primary breast implant or tissue expander between January 1, 2015 and December 31, 2019. Indications for a breast implant or tissue expander were breast augmentation or reconstruction. Patients who had received any previous breast implant surgery or in whom additional surgical techniques had been used during implant insertion (ADM/Mesh, fat grafting, autologous flap cover, or mastopexy) were excluded from the analysis to reduce group heterogeneity. Patients with missing data about the use of ICMs were also excluded (Supplemental Figure 1).

### Data collection: the Dutch Breast Implant Registry

The Dutch Breast Implant Registry (DBIR) is a national, population-based registry, for which data collection started in 2015. For each inserted or explanted implant, information on patient, surgery, and implant characteristics are collected. In the Netherlands, breast implant surgery is performed in both hospitals and private clinics. Currently, 74 hospitals (100%) and 37 private clinics (95%), where breast implant surgery is being performed, actively register breast implants and tissue expanders in DBIR. Data are securely stored at a Dutch Trusted Third Party for medical data (MRDM), according to the General Data Protection Regulation (GDPR). More details about the registry have been published previously.^27^, ^28^, ^29^ For the current study, we used the data as updated on May 8, 2020.

### Definitions

Primary implant insertion was defined as the first operation known in the registry in which a new breast implant or tissue expander was inserted. Breast augmentation was defined as a surgical intervention to enlarge breasts for cosmetic reasons. Breast reconstruction included reconstructions after mastectomy due to breast cancer, after risk-reducing mastectomy, or after benign conditions such as a congenital deformity, tuberous breasts, or gender-affirming surgery. Seven prospectively collected infection control measures (ICMs) were evaluated: preoperative prophylactic antibiotics, implant and/or pocket irrigation with betadine and/or an antibiotic solution, change of gloves before implant handling, nipple guards, use of an insertion sleeve such as the Keller funnel, postoperative drains, and postoperative prophylactic antibiotics. Per implant, the total number of ICMs used at implant insertion was calculated and classified as lower than the median number, equal to, or higher than the median number of ICMs. Infection-related revision surgery was defined as the first reoperation after insertion, in which the implant or expander was repositioned, explanted, or replaced due to an infection. Exact definitions of all variables used for analysis are based on the ICOBRA (International Collaboration of Breast Registry Activities) core dataset and can be found in the data dictionary (Supplemental Table 1).^30^

### Outcomes

The outcomes were the number and combinations of ICMs used per primary implant insertion, the infection-related cumulative revision incidence over time in relation to the number of ICMs used, and the variation between Dutch healthcare institutions in the use of each ICM.

### Statistical analysis

All analyses were performed separately for breast augmentations and reconstructions, with the implant as the unit of analysis because most of the ICMs were applied per implant side, and each implant has a risk of revision surgery.

Missing data patterns were evaluated, thus resulting in the assumption of data being missing at random. Multiple imputation by chained equations was performed, thus creating 10 imputed datasets with 50 iterations each, by using all variables, including the outcome variable (“mice” package version 3.11.0).^31^^,^^32^ In the case of highly correlated variables, only one was used as the auxiliary variable. The outcome variable itself was not imputed. Statistical models were fitted, and results were pooled following the Rubin's rules.^33^ See Supplementary Table 2 for non-imputed data.

Baseline characteristics were compared between the groups with less than, equal, or more than the median number of ICMs used per primary implant insertion. Student's t-tests were performed for continuous variables and Chi-square tests or Fisher's exact tests for categorical variables. Reported *P*-values are two-sided, and values <0.05 were considered statistically significant.

The crude, infection-related cumulative revision incidence was calculated for both indication groups using the Fine-Gray cumulative incidence function.^34^ Scheduled replacements of tissue expanders for breast implants and unplanned revisions due to other indications were included as competing risks. Subsequently, we compared the infection-related cumulative revision incidence between the three ICM groups using a log-rank test. Implants without any revision at closure of the dataset on May 8, 2020 were censored.

The variation between healthcare institutions in the use of each ICM was calculated for the years 2015-2019 and are visualized in Sina plots, including nationwide means and 95% confidence intervals.

All analyses were performed with R software, version 1.3.959 – © 2009-2020, RStudio, Inc.

## Results

From 2015 to 2019, 28,653 patients and 52,415 implants met the inclusion criteria (Supplementary Figure 1). Of these, 44,474 implants (84.8%) were inserted for breast augmentation and 7,941 (15.2%) for breast reconstruction. The procedures were performed in 114 healthcare institutions, including university or specialized (breast) cancer hospitals, general hospitals, and private clinics.

### Number of infection control measures used

**Figure 1:**
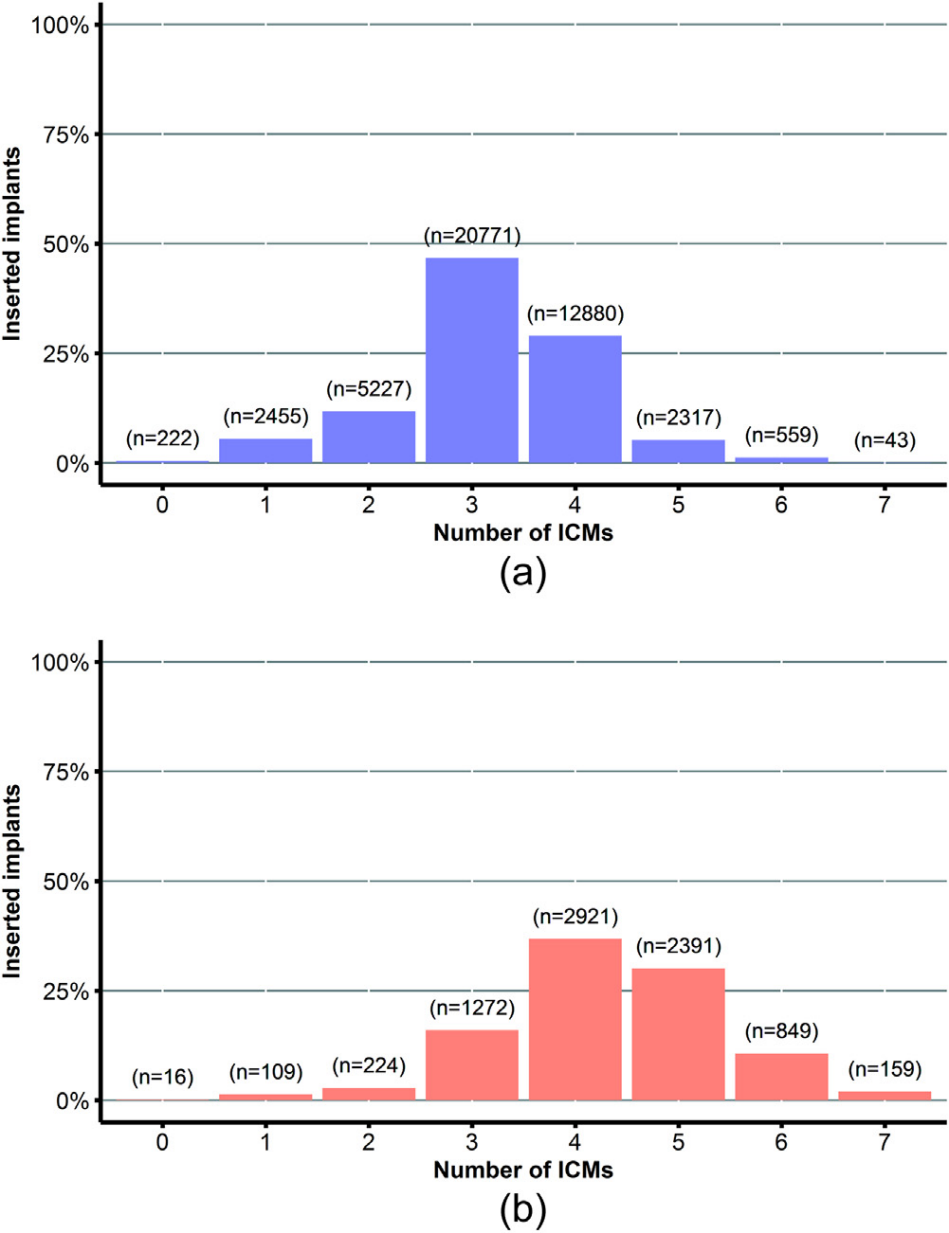
Number of infection control measures used per primary implant insertion. **A.** Breast augmentation **B.** Breast reconstruction *ICMs, infection control measures.*

For breast augmentation, the median number of ICMs used per primary implant insertion was 3 (IQR 3-4). Of the 44,474 primary augmentation procedures, 17.8% (n=7,904) were performed with fewer than three ICMs, 46.7% (n=20,771) with three ICMs, and 35.5% (n=15,799) with more than three ICMs (Figure 1A). These three ICM groups showed statistically significant differences in all baseline characteristics (Table 1).

**Table 1:** Patient, surgery, and implant characteristics per number of infection control measures used, per primary implant insertion.

	Breast reconstruction				Breast augmentation			
	<4 ICMs (<median, *n* = 1621)	4 ICMs (median, *n* = 2921)	>4 ICMs (>median, *n* = 3399)	*P*	<3 ICMs (<median, *n* = 7904)	3 ICMs (median, *n* = 20771)	>3 ICMs (>median, *n* = 15799)	*P*
*Patient characteristics*								
Age in years*	47.6 (12.2)	47.6 (12.0)	48.5 (11.6)	0.003	31.0 (9.0)	30.8 (8.9)	32.3 (9.9)	<0.001
**ASA classification**				<0.001				<0.001
I	1191 (73.4)	1934 (66.2)	2100 (61.8)		7435 (94.1)	20118 (96.9)	14378 (91.0)	
II	385 (23.8)	907 (31.1)	1185 (34.8)		464 (5.9)	640 (3.1)	1368 (8.7)	
III+	45 (2.8)	80 (2.7)	114 (3.4)		5 (0.1)	13 (0.1)	53 (0.3)	
**Body mass index in kg/m^2^†**	23.5 (21.3-26.4)	23.8 (21.2-26.9)	24.1 (21.6-27.0)	<0.001	21.6 (19.4-24.1)	21.6 (19.7-23.9)	21.4 (19.6-23.5)	<0.001
**Smoking status**				0.820				<0.001
Not smoking	1427 (88.0)	2566 (87.8)	3003 (88.3)		6672 (84.4)	16758 (80.7)	12591 (79.7)	
Smoking	194 (12.0)	355 (12.2)	396 (11.7)		1232 (15.6)	4013 (19.3)	3208 (20.3)	
**Previous radiotherapy**				0.007				<0.001
No	1509 (93.1)	2774 (95.0)	3225 (94.9)		7864 (99.5)	20739 (99.8)	15757 (99.7)	
Yes	112 (6.9)	147 (5.0)	174 (5.1)		40 (0.5)	32 (0.2)	42 (0.3)	
*Surgery characteristics*								
**Year of surgery**				<0.001				<0.001
2015	298 (18.4)	547 (18.7)	435 (12.8)		1780 (22.5)	2204 (10.6)	1429 (9.1)	
2016	312 (19.2)	716 (24.5)	728 (21.4)		2576 (32.6)	3563 (17.2)	2437 (15.4)	
2017	283 (17.5)	687 (23.5)	738 (21.7)		1784 (22.6)	5053 (24.3)	3182 (20.1)	
2018	295 (18.2)	511 (17.5)	749 (22.0)		915 (11.6)	4926 (23.7)	4329 (27.4)	
2019	433 (26.7)	460 (15.7)	749 (22.0)		849 (10.7)	5025 (24.2)	4422 (28.0)	
**Healthcare institution volume per year**				<0.001				<0.001
<100 implant surgeries	430 (26.5)	891 (30.5)	1156 (34.0)		509 (6.5)	594 (2.9)	1401 (8.9)	
100-249 implant surgeries	626 (38.6)	1536 (52.6)	1774 (52.2)		1147 (14.5)	2298 (11.1)	3263 (20.6)	
250-500 implant surgeries	297 (18.3)	456 (15.6)	437 (12.9)		2130 (26.9)	4250 (20.5)	5230 (33.1)	
>500 implant surgeries	268 (16.5)	38 (1.3)	32 (0.9)		4118 (52.1)	13629 (65.5)	5905 (37.4)	
**Laterality**				<0.001				<0.001
Unilateral	996 (61.4)	1606 (55.0)	2030 (59.7)		34 (0.4)	53 (0.3)	175 (1.1)	
Bilateral	625 (38.6)	1315 (45.0)	1369 (40.3)		7870 (99.6)	20718 (99.7)	15624 (98.9)	
**Incision site**				<0.001				<0.001
Inframammary	298 (18.4)	377 (12.9)	488 (14.4)		7726 (97.7)	20503 (98.7)	15562 (98.5)	
Mastectomy scar	1109 (68.4)	2112 (72.3)	2517 (74.1)		94 (1.2)	106 (0.5)	91 (0.6)	
Areolar	129 (8.0)	278 (9.5)	278 (8.2)		23 (0.3)	33 (0.2)	56 (0.4)	
Other	85 (5.2)	154 (5.3)	116 (3.4)		61 (0.8)	129 (0.6)	90 (0.5)	
**Plane**				<0.001				<0.001
Subglandular	0 (0)	0 (0)	0 (0)		1150 (14.5)	2604 (12.5)	2813 (17.8)	
Subcutaneous or subfascial	154 (9.5)	145 (5.0)	211 (6.2)		241 (3.0)	2828 (13.6)	539 (3.4)	
Completely covered with PM	874 (53.9)	1809 (61.9)	2176 (64.0)		2229 (28.2)	2807 (13.5)	2011 (12.7)	
Partially covered with PM	593 (36.6)	967 (33.1)	1012 (29.8)		4284 (60.3)	12532 (60.4)	10436 (66.1)	
*Implant characteristics*								
**Inserted implant type**				<0.001				<0.001
Breast implant	847 (52.3)	904 (30.9)	815 (24.0)		7883 (99.7)	20737 (99.8)	15738 (99.6)	
Tissue expander	774 (47.7)	2017 (69.1)	2584 (76.0)		21 (0.3)	34 (0.2)	61 (0.4)	
**Texture**				<0.001				<0.001
Textured	1523 (94.0)	2838 (97.2)	3249 (95.6)		6830 (86.4)	19769 (95.2)	13768 (87.1)	
Smooth	57 (3.5)	21 (0.7)	32 (0.9)		179 (2.3)	770 (3.7)	1812 (11.5)	
Polyurethane	41 (2.5)	62 (2.1)	118 (3.5)		895 (11.3)	232 (1.1)	219 (1.4)	

Values in parentheses are percentages unless indicated otherwise; values are *mean (SD) and †median (IQR). ICMs, infection control measures; ASA, American society of anesthesiologists; PM, pectoralis major.

For breast reconstruction, the median number of ICMs used per primary implant insertion was 4 (IQR 4-5). Of the 7,941 primary implant procedures, 20.4% (n=1,621) were performed with fewer than four ICMs, 36.8% (n=2,921) with four ICMs, and 42.8% (n=3,399) with more than four ICMs (Figure 1B)*.* Among these three ICM groups, statistically significant differences were seen in most baseline characteristics (Table 1).

### Combinations of infection control measures

Most surgeons who only used one ICM during breast augmentation used only preoperative antibiotics (73.1%). If more than one ICM was used, preoperative antibiotics were usually combined with implant and/or pocket irrigation, followed by the change of gloves, nipple guards, drains, postoperative antibiotics, and an insertion sleeve (in this exact order).

Most surgeons who only used one ICM during breast reconstruction used only postoperative drains (64.2%). If more than one ICM was used, postoperative drains were most frequently combined with preoperative antibiotics, followed by the change of gloves, implant and/or pocket irrigation, postoperative antibiotics, nipple guards, and an insertion sleeve (in this exact order).

### Cumulative incidence of infection-related revisions

**Figure 2:**
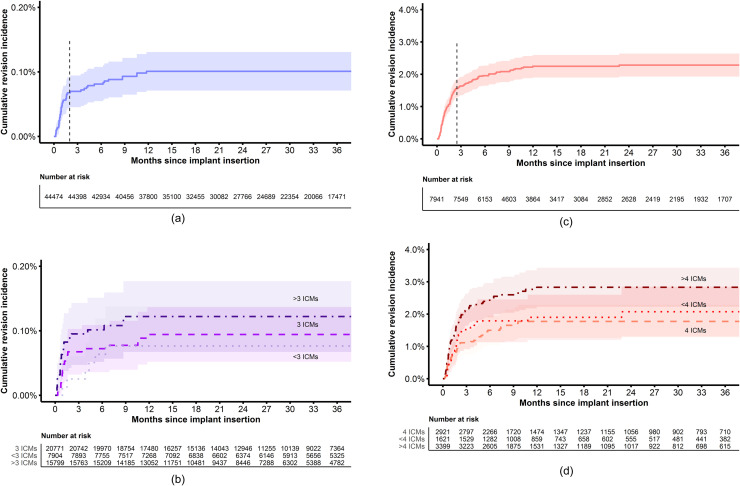
Crude cumulative revision incidence of infection-related revisions since primary implant insertion. **A.** Breast augmentation (total group) **B.** Breast augmentation (per ICM group)† **C.** Breast reconstruction (total group) **D.** Breast reconstruction (per ICM group)* Different scales on y-axis. The dotted vertical line represents the time in which most infection-related revisions occurred (augmentation: 2 months, reconstruction: 2.5 months). ICM, infection control measure. Log-rank test: †P = 0.500 and *P = 0.020.

For the total breast augmentation group, the median follow-up was 30.1 (IQR 17.2-44.4) months. The crude cumulative incidence of infection-related revisions was 0.1% within 3 years (95% CI 0.1-0.1) (Figure 2A). Most infection-related revisions occurred within 2 months. The cumulative incidence of infection-related revisions between the three ICM groups was comparable, with <0.1% (95% CI 0-0.1) for less than three ICMs, 0.1% (95% CI 0.1-0.1) for three ICMs, and 0.1% (95% CI 0.1-0.2) for more than three ICMs (*P* = 0.500) (Figure 2B).

For the total breast reconstruction group, the median follow-up was 33.7 (IQR 18.6-47.1) months. The crude cumulative incidence of infection-related revisions was 2.1% (95% CI 1.8-2.4) within 3 years (Figure 2C). Most infection-related revisions occurred within 2.5 months. Paradoxically, the cumulative incidence of infection-related revisions differed between the three ICM groups, with 2.1% (95% CI 1.3-2.8) for less than four ICMs, 1.8% (95% CI 1.3-2.3) for four ICMs, and 2.8% (95% CI 2.2-3.4) for more than four ICMs (*P* = 0.020) (Figure 2D).

The association between the number of ICMs used and the infection-related revision incidence could not be studied further for both indication groups. The association between different combinations of ICMs and the infection-related revision incidence could also not be studied . Event rates were too low to appropriately adjust for confounding factors and to limit confounding by indication.

### Nationwide variation

**Figure 3:**
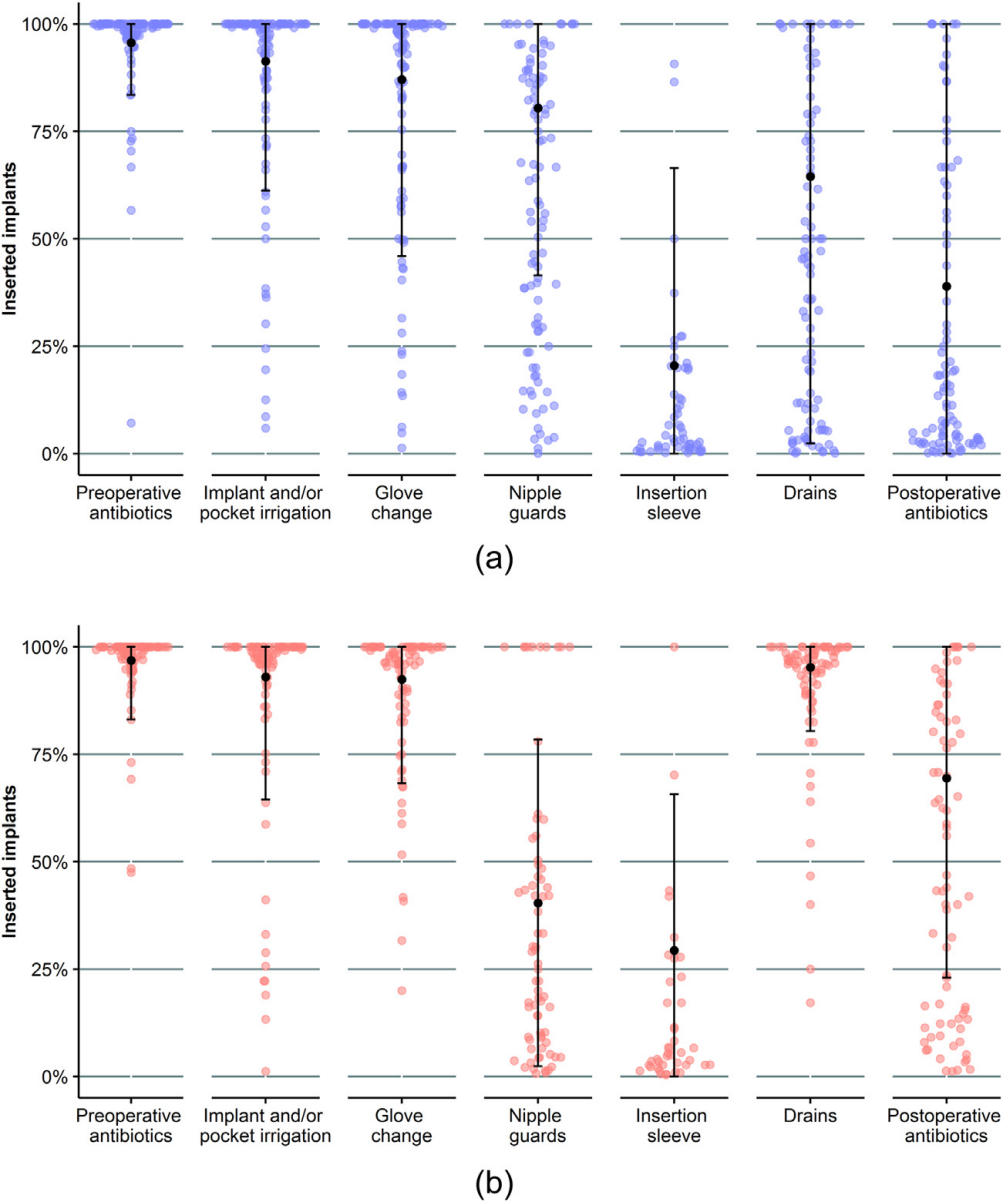
Nationwide variation in the proportion of implants being inserted with a particular infection control measure. **A.** Breast augmentation **B.** Breast reconstruction *The 95% confidence intervals are displayed around the nationwide mean in black.*

For breast augmentations, the four most frequently used ICMs were preoperative antibiotics (nationwide mean 95.6%, 95% CI 83.4-100), implant and/or pocket irrigation (91.3%, 61.2-100), glove change (87.1%, 45.9-100), and nipple guards (80.4%, 41.4-100) (Figure 3A). Between the institutions, most variation was seen in the use of drains (nationwide mean 64.5%, 95% CI 2.4-100), postoperative antibiotics (38.9%, 0-100), and an insertion sleeve (20.5%, 0-66.5).

For reconstructive indications, the four most frequently used ICMs were preoperative antibiotics (nationwide mean 96.8%, 95% CI 83.1-100), drains (95.2%, 80.4-100), implant and/or pocket irrigation (93.0%, 64.4-100), and glove change (92.4%, 68.2-100) (Figure 3B). Between the institutions, most variation was seen in the use of postoperative antibiotics (nationwide mean 69.4%, 95% CI 22.9-100), nipple guards (40.4%, 2.4-78.4), and an insertion sleeve (29.3%, 0-65.7).

## Discussion

This nationwide population-based study included registry data from almost all healthcare institutions performing breast implant surgery in the Netherlands. For breast augmentation, most surgeons used three different infection control measures (ICMs), and for breast reconstruction, four ICMs were used. The infection-related revision incidence was low (augmentation: 0.1%, reconstruction: 2.1%) and most infection-related revisions occurred within 2 and 2.5 months after breast augmentation and reconstruction, respectively. These numbers are in line with current literature.^9^^,^^10^^,^^12^ The use of different ICMs varied considerably between institutions.

Unfortunately, the impact of the number and combinations of ICMs used on the infection-related revision incidence remained unclear because it appeared statistically impossible to properly adjust for confounding factors and limit confounding by indication due to the low event rate. Therefore, paradoxically it seemed that implants inserted with more than four ICMs during breast reconstruction had a higher chance of infection-related revision. However, it is more likely that this was due to higher patient age, ASA classification, and BMI, known as confounding by indication. If proper adjustment for these baseline characteristics would have been possible, the found effect would probably disappear.

The use of more ICMs during breast reconstruction than that during breast augmentation supports the hypothesis that more ICMs are used in higher-risk patients. However, in a post hoc analysis, no consistent association was found between high-risk baseline characteristics and the use of more ICMs (data not shown). Therefore, another explanation is more likely. In the Netherlands, most healthcare institutions have an infection prevention unit, which provides strict protocols based on (inter)national guidelines.^18^ As a result, most surgeons have developed a standard practice for all patients, using at least the ICMs prescribed by their local protocol. In some specific situations, possibly one or two additional ICMs are used. For example, the use of postoperative antibiotic prophylaxis if surgery lasted substantially longer than expected, or the use of a Keller funnel if polyurethane-coated implants were inserted. However, it is unlikely that surgeons use fewer ICMs than advised by their infection prevention protocols, regardless of the patient's risk profile.

These institutional infection prevention protocols could also explain the considerable variation between Dutch healthcare institutions in the use of each ICM. Most guidelines and studies have reported the potential beneficial use of preoperative antibiotic prophylaxis, glove change, implant and/or pocket irrigation, and in the case of breast reconstruction, the use of drains. However, to the best of our knowledge, no high-level evidence has been obtained proving the advantage or disadvantage of postoperative prophylactic antibiotics, nipple guards, or insertion sleeves.^18^, ^19^, ^20^, ^21^^,^^35^^,^^36^ Therefore, local protocols may vary, thus resulting in nationwide variation.

Additionally, some surgeons might consistently use more ICMs than proven in literature because of the hypothesized bacterial aetiology of breast implant-associated anaplastic large cell lymphoma (BIA-ALCL) and capsular contracture.^24^^,^^29^^,^^37^^,^^38^ A non-evidence-based, so-called 14-point plan was proposed by Deva et al. in 2013 to stimulate using 14 steps to reduce implant infections and hypothetically BIA-ALCL.^38^ These developments may have caused surgeons to practice more defensive medicine over the years, as was evident from our data showing increased use of ICMs in breast reconstruction and breast augmentation procedures.

For now, however, it remains unclear how the use of each ICM and the variation in current practice impact the quality of care provided. Although this study included data from more than 52,000 implants, infection-related revision incidence was too low to allow for statistical testing of differences, appropriately adjusting for confounding factors, limiting confounding by indication, and considering the various combinations of ICMs. However, the low infection-related revision incidence could also reflect high-quality standards. Further reduction of infection-related revisions may therefore be difficult to achieve. Studying this in even larger study populations may also mean that even if differences would become statistically significant, they may not necessarily be clinically relevant. Therefore, one might even explore reducing the number of non-evidence-based ICMs to reduce costs and variation, although investigating the underlying reasons for the observed variation may be an important next step for improving guidelines and the quality of care for patients in both cosmetic and reconstructive breast implant surgery.

### Strengths and limitations

This is the first study investigating the national variation in the use of seven commonly used ICMs, using prospectively collected real-world data from a nationwide population-based registry, including both breast augmentations and reconstructions. Consequently, our findings reflect daily clinical practice in all sorts of hospitals and private clinics in the Netherlands. Additionally, infection-related revisions beyond 30 postoperative days were considered because a substantial proportion of infections occur after 30 days.^12^ Finally, the DBIR uses definitions similar to all breast implant registries affiliated with the International Collaboration of Breast Registry Activities (ICOBRA).^30^ This improves comparability and probability of data pooling for future studies that use data from these affiliated registries.

However, this study is not exempt from limitations. Registration of all inserted and explanted breast implants in DBIR is mandatory for board-certified plastic surgeons. The registration of inserted implants can be externally validated using sales data, for example. The external validation of explanted implants, however, is more difficult as reliable tools are unavailable. Therefore, revision surgeries might have been underreported without us knowing. Although the presented revision incidences are in line with current literature, they need to be interpreted as a minimum incidence. Second, reconstructions after non-nipple sparing mastectomy are not eligible for nipple guards. However, in DBIR, the distinction between nipple-sparing vs non-nipple-sparing mastectomy has not been registered between 2015 and 2019. Therefore, the actual proportion of nipple guards used in breast reconstruction after a nipple-sparing mastectomy might be higher than presented in this study.

## Conclusion

Although the use of different ICMs varied considerably between institutions, the incidence of infection-related revisions after breast implant surgery was generally low. Most infection-related revisions occurred within 2–2.5 months. Most surgeons used three ICMs for breast augmentation and four ICMs for breast reconstruction. As it remains unclear how the variation in ICM use impacts the quality of care provided, further investigation of the underlying reasons is needed.

## Declaration of Competing Interest

None.

## Appendix Supplementary materials

Image, application 1

Image, application 2
