# Supplementary material for: Variation in the use of infection control measures and infection-related revision incidence after breast implant surgery in the Netherlands
Source: JPRAS Open. 2022 Oct 12;34:226–38. doi: 10.1016/j.jpra.2022.10.004 (PMC9713279; doi:10.1016/j.jpra.2022.10.004)
Supplement: Supplementary file 1 [file mmc1.docx]

**
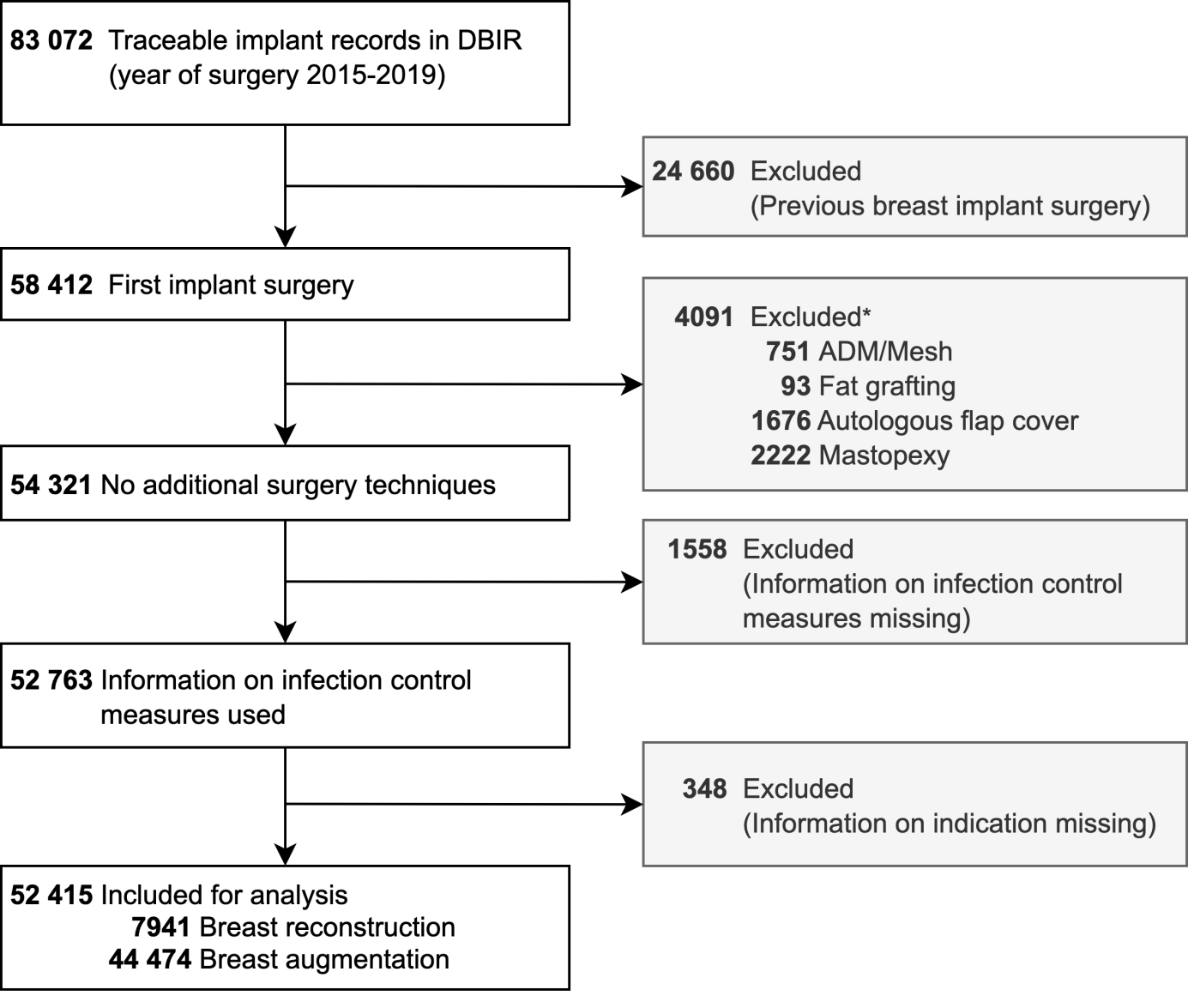
**

**Supplemental Figure 1. Inclusion criteria.**

**More than one additional surgery technique could be registered per record. DBIR, Dutch Breast Implant Registry; ADM, acellular dermal matrix.*

**Supplemental Table 1. Definitions of the Variables Used for this Study, derived from the ICOBRA Harmonized Dataset.**

| **LABEL** | **VARIABELE AS STATED IN DBIR** | **DEFINITION** |
| --- | --- | --- |
| age | calculated manually | Calculated in years, using the date of surgery and date of birth. |
| ASA classification | surgasa | American Society of Anesthesiologists' identification system for a patient's medical status. |
| body mass index | bmi | Is calculated automatically when height (in centimeters) and weight (in kilograms) of the patient are filled in. |
| smoking status | nicotineabuse | As identified by the patient. |
| previous radiotherapy | prevradio | Radiotherapy to the breast or chest wall at any time prior to the current device operation. |
| year of surgery | calculated manually | Derived from the variable [operdate]. |
| indication | indication | The reason for surgery:  - Cosmetic augmentation = Cosmetic surgery for enlarging breasts. - Reconstruction post mastectomy for cancer = Surgery to recreate a breast after one or both breasts are removed as a treatment for breast cancer. - Reconstruction post prophylactic mastectomy = Surgery to remove one or both breasts to reduce the risk of developing breast cancer. - Reconstruction benign = Surgery to restore or create shape and symmetry in patients with loss or absence of all or some breast tissue due to benign breast conditions, congenital deformity, tuberous breast, or gender reassignment surgery. |
| first implant surgery | first | Yes = Primary surgery: initial insertion of a new device, i.e. an implant or tissue expander. No = Insertion of a new device in a patient who has had previous breast implant surgery. |
| healthcare institution volume | calculated manually | Calculated per healthcare institution, per year, by counting the total number of implant procedure records (regardless of the operation indication). |
| laterality | surgside | The left and/or right breast. |
| number of infection control measures (ICMs) used | calculated manually | Counted the number of applied Infection Control Measures using the variables [systantibiot = Use of antibiotics I.V. before incision], [postopantibiot = Use of antibiotics I.V. at any time after 3 hours post-surgery], [antisepticrinse = Rinse of the surgically created pocket or implant before implant insertion with antiseptic solution, antibiotic solution or both], [kellerfunnel = A skin barrier protector such as a Keller funnel], [nippleguards = The use of adhesive film dressing covering the nipple-areola complex to prevent perioperative expression of bacteria from nipple ducts contaminating the operative field. In case of no nipples: no nipple guards], [glovchanginsert = Change of gloves immediately prior to insertion of the implant], [drains = use of drains]. |
| incision site | incissite | The site where the incision is placed: - Inframammary: An incision in, or beneath, the infra-mammary fold. - Mastectomy scar: An incision through an existing mastectomy incision (nipple-sparing or non-nipple sparing). - Areolar: An incision around the areola. - Other: Any other incision site, such as an axillary incision. |
| plane | plane | The surgical plane in which an implant will be inserted:  - Sub glandular: Beneath the gland and above the muscular fascia. - Subcutaneous or subfascial: Directly beneath the subcutis or above the pectoralis muscle but below the pectoralis fascia. - Completely covered with PM: Above chest wall & below the pectoralis major muscle - Partially covered with PM: Partially beneath pectoralis major muscle and breast parenchyma. |
| ADM/Mesh | sheet | The use of either an:  - ADM: acellular dermal matrix - Mesh: absorbable or non-absorbable synthetic mesh.  Both are medical devices used in breast implant surgery where the mesh or matrix provides a soft tissue scaffold. |
| fat grafting | fatgrafting | Transfer of aspirated fat to the breast region. |
| autologous flap cover | calculated manually | If either the variable [ldcover = Latissumus Dorsi flap used for current breast implant surgery that covers an implantable breast device or adds volume to the breast mound] or [flapcoverother = Any other flap used for current breast implant surgery that covers an implantable breast device or adds volume to the breast mound] was selected. |
| mastopexy | mastopexy | Breast lift. |
| implant type | implanttype | Permanent breast implant or Tissue Expander. |
| texture | calculated manually | Combination of the variable [texture = The surface texture of the device being inserted or explanted] and [coating = Breast implants with a polyurethane coating are Polytech and Silimed for example] |
| infection-related revision | wound | revision due to an infection associated with a breast implant which leads to its explantation or revision. Usually involves redness, localized pain or tenderness, abscess or persistent serous liquid formation around the implant. |
| **An extended version of the data dictionary can be downloaded from https://support.mrdm.nl/documentatie/* | | |

**Supplemental Table 2. Raw Data of Patient, Surgery, and Implant Characteristics at Time of Primary Implant Insertion.**

|  | **Breast Reconstruction**  (*n* = 7941) |  | **Breast Augmentation**  (*n* = 44474) |
| --- | --- | --- | --- |
| *Intervention of Interest* | | | |
| **Number of ICMs (median, IQR)** | 4 (4-5) |  | 3 (3-4) |
| Missing | 0 (0) |  | 0 (0) |
| *Patient Characteristics* | | | |
| **Age in years (mean, SD)** | 48.0 (11.9) |  | 31.4 (9.3) |
| Missing | 28 (0.4) |  | 235 (0.5) |
| **ASA classification** |  |  |  |
| I | 5153 (64.9) |  | 41739 (93.8) |
| II | 2447 (30.8) |  | 2450 (5.5) |
| III+ | 237 (3.0) |  | 70 (0.2) |
| Missing | 104 (1.3) |  | 215 (0.5) |
| **Body mass index** **in** **kg/m^2^ (median, IQR)*** | 24.0 (21.8-26.9) |  | 21.5 (20.1-23.4) |
| Missing | 295 (7.9) |  | 1229 (5.3) |
| **Smoking status*** |  |  |  |
| Not smoking | 2570 (68.7) |  | 12400 (53.3) |
| Smoking | 393 (10.5) |  | 3205 (13.8) |
| Missing | 779 (20.8) |  | 7676 (32.9) |
| **Previous radiotherapy** |  |  |  |
| No | 6470 (81.5) |  | 43143 (97.0) |
| Yes | 379 (4.8) |  | 96 (0.2) |
| Missing | 1280 (13.7) |  | 1235 (2.8) |
| *Surgery Characteristics* | | | |
| **Year of surgery** |  |  |  |
| 2015 | 1280 (16.1) |  | 5413 (12.2) |
| 2016 | 1756 (22.1) |  | 8576 (19.3) |
| 2017 | 1708 (21.5) |  | 10019 (22.5) |
| 2018 | 1555 (19.6) |  | 10170 (22.9) |
| 2019 | 1642 (20.7) |  | 10296 (23.1) |
| Missing | 0 (0) |  | 0 (0) |
| **Healthcare institution volume per year** |  |  |  |
| <100 implant surgeries | 2477 (31.2) |  | 2504 (5.6) |
| 100-249 implant surgeries | 3936 (49.5) |  | 6708 (15.1) |
| 250-500 implant surgeries | 1190 (15.0) |  | 11610 (26.1) |
| >500 implant surgeries | 338 (4.3) |  | 23652 (53.2) |
| Missing | 0 (0) |  | 0 (0) |
| **Laterality** |  |  |  |
| Unilateral | 4632 (58.3) |  | 262 (0.6) |
| Bilateral | 3309 (41.7) |  | 44208 (99.3) |
| Missing | 0 (0) |  | 4 (<0.1) |
| **Incision site** |  |  |  |
| Inframammary | 970 (12.2) |  | 43703 (98.3) |
| Mastectomy scar | 5341 (67.3) |  | 245 (0.6) |
| Areolar | 647 (8.1) |  | 106 (0.2) |
| Other | 310 (3.9) |  | 280 (0.6) |
| Missing | 673 (8.5) |  | 140 (0.3) |
| **Plane** |  |  |  |
| Subglandular | 0 (0) |  | 6461 (14.5) |
| Subcutaneous or subfascial | 428 (5.5) |  | 3579 (8.1) |
| Completely covered with PM | 4431 (55.7) |  | 6850 (15.4) |
| Partially covered with PM | 2162 (27.2) |  | 26911 (60.5) |
| Missing | 920 (11.6) |  | 673 (1.5) |
| *Implant Characteristics* | | | |
| **Inserted implant type** |  |  |  |
| Breast implant | 2566 (32.3) |  | 44358 (99.7) |
| Tissue expander | 5375 (67.7) |  | 116 (0.3) |
| Missing | 0 (0) |  | 0 (0) |
| **Texture** |  |  |  |
| Textured | 7005 (88.2) |  | 39822 (89.5) |
| Smooth | 101 (1.3) |  | 2739 (6.2) |
| Polyurethane | 195 (2.5) |  | 1321 (3.0) |
| Missing | 640 (8.1) |  | 592 (1.3) |
| *Values in parentheses are percentages unless indicated otherwise. ICM, infection control measure; IQR, interquartile range; SD, standard deviation; ASA, American society of anesthesiologists; PM, pectoralis major. *Registered since September 2017 and therefore presented for a smaller population (reconstruction: n=3742, augmentation: n=23281).* | | | |
