# Supplementary figures and images for: Variation in the use of infection control measures and infection-related revision incidence after breast implant surgery in the Netherlands
Source: JPRAS Open. 2022 Oct 12;34:226–38. doi: 10.1016/j.jpra.2022.10.004 (PMC9713279; doi:10.1016/j.jpra.2022.10.004)

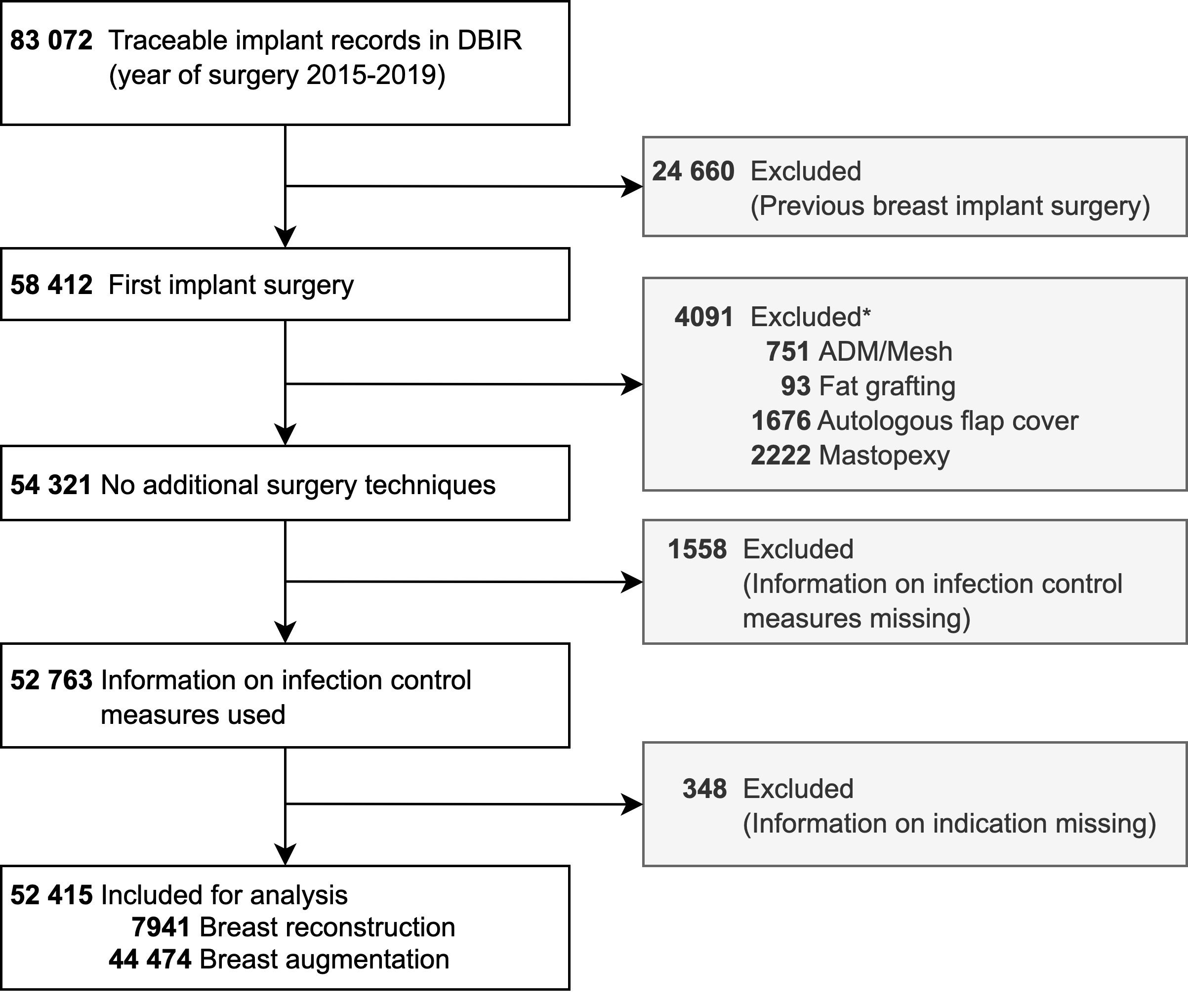

Supplement: Supplementary file 2 [file mmc2.zip › mmc2.png]
